# The Enrichment of Acetic Acid Using an Integrated Reverse Osmosis–Electrodialysis Process

**DOI:** 10.3390/membranes15050129

**Published:** 2025-04-27

**Authors:** Shichang Xu, Long Zhang, Zhen Zhang, Lixin Xie, Wen Zhang

**Affiliations:** 1 State Key Laboratory of Chemical Engineering, School of Chemical Engineering and Technology, Tianjin University, Tianjin 300350, China; xu_sc1@tju.edu.cn (S.X.); zhanglong123@tju.edu.cn (L.Z.); zhangzhen123@tju.edu.cn (Z.Z.); 2 Tianjin Key Laboratory of Membrane Science & Desalination Technology, School of Chemical Engineering and Technology, Tianjin University, Tianjin 300350, China

**Keywords:** acetic acid, enrichment, reverse osmosis, electrodialysis, integrated process

## Abstract

In this study, the integrated process of reverse osmosis (RO) and electrodialysis (ED) is developed to concentrate the dilute solution of acetic acid (HAc). The key parameters, such as RO pressure, ED voltage, and ED volume ratio, were systematically evaluated and the operation conditions of the processes were optimized. Under an operating pressure of 5 MPa, RO can enrich low-concentration HAc from 1.5 wt.% to 6.5% wt.% and the energy consumption is 0.37 kW·h·kg^−1^. Next, RO-concentrated water was used as the ED feed and the first ED with a volume ratio of the concentrated to dilute chamber of 1:4 was carried out under the conditions of a flow rate of 30 L/h and an operating voltage of 12 V; the HAc concentration reached 12.50 wt.%. The second ED with a volume ratio of 1:5 made the final HAc concentration reach 19.02 wt.%. This study shows that using RO-concentrated water instead of initial water for the ED process can reduce water energy consumption and cost markedly, and the RO–ED integrated process can efficiently pre-enrich low-concentration HAc aqueous solution, and the enriched HAc concentration meets the requirements for the further distillation of HAc.

## 1. Introduction

Dilute HAc aqueous solutions have been produced in several industries, posing significant environmental and ecosystem risks [1,2,3,4]. Distillation is the conventional method for separating acetic acid from aqueous solutions [5,6,7]. However, distillation is used to separate a large amount of medium- and high-concentration HAc aqueous solutions in industry. Even if HAc is recovered by extractive distillation and reactive distillation, the initial HAc feed solution concentration is usually greater than 20 wt.% [6]. Therefore, more efficient strategies need to be developed to concentrate HAc from low to high concentration to meet the demand for distillation.

Membrane separation is an alternative technology for acid enrichment and has demonstrated advantages such as appropriate energy consumption, sustainable processing, and relatively straightforward scalability [8,9]. Several membrane separation processes, including nanofiltration [10,11,12] (NF), reverse osmosis [11,12,13,14] (RO) driven by pressure, pervaporation [15,16] (PV) driven by partial pressure differences, and electrodialysis [17,18,19,20] (ED) driven by voltage, have received considerable attention for the separation and purification of HAc. Compared with RO, NF usually suffers from high energy consumption [13,14]. In the PV process using silicalite membranes, the permeate contains an almost constant HAc concentration, indicating low HAc selectivity [21].

RO boasts benefits such as lower energy consumption, cost-effectiveness, and seamless integration [14,22]. However, it is rarely used to treat low-concentration aqueous acidic solutions. ED emerges as an alternately advantageous membrane separation technique, driven by an electric field and reliant on ion-exchange membranes as a centerpiece [23]. ED is favored due to its cost efficiency, high separation precision, minimal environmental footprint, and operational simplicity. Moreover, ED has been reported to offer a great advantage of achieving a high acid concentration. Despite these benefits, ED energy consumption is high, especially when the concentration is very low [24]. Therefore, integrating RO and ED processes may restrain the shortcoming of high energy consumption in ED to develop an efficient strategy for the separation processes. For example, in the treatment of industrial lithium-containing wastewater using an integrating RO–ED process, the use of RO retentate instead of initial wastewater in the ED process can reduce the water energy consumption and cost markedly, and the hybrid RO–ED process allows for lithium salt extraction and concentrating from industrial lithium-containing wastewater, which is appropriate for industrial applications [24]. Hence, the integrating RO–ED process may also provide a clean and efficient strategy for the enrichment of HAc solution.

Hence, in this study, we utilized an integrated strategy combining RO with ED to enrich aqueous HAc solutions. The conditions in the RO–ED processes were optimized and a comparative analysis was also conducted to evaluate the energy consumption.

## 2. Experiment

### 2.1. Materials

Acetic acid and oxalate (99.5% Tianjin Kemel Co., Ltd., Tianjin, China) and ethanol absolute solution (water content < 0.005%, Aladdin Reagent Co., Ltd., Shanghai China) were used in the experiments. All the water used in this study was DI water (conductivity < 10 µS·cm^−1^). The RO membrane was purchased from Dow Chemical Co., Ltd., Houston, TX, USA and the cation exchange membranes (CEM) and anion exchange membranes (AEM) were purchased from Lanran Co., Ltd., Hangzhou, China. The electrode membranes (EM) were the perfluorosulfonic acid cation exchange membranes purchased from Dongyue Group Co., Ltd, Zibo, China. The main properties of the RO membrane are shown in Table 1. The main properties of CEM and AEM are shown in Table 2. The main properties of EM are shown in Table 3.

### 2.2. RO Experiment

**Figure 1:**
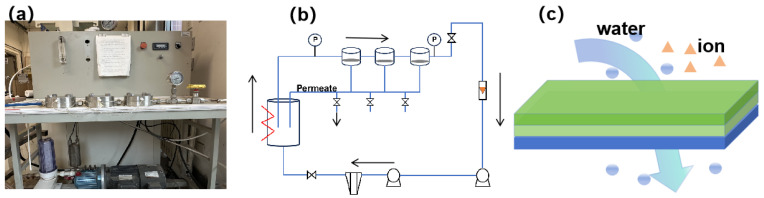
The experimental equipment of RO. (**a**) The schematic diagram of lab-scale RO configuration (**b**) and RO mechanism (**c**).

The laboratory device is designed and assembled of RO as shown in Figure 1. The unit of the RO process consisted of a membrane cell, a flow meter, a manometer, a high-pressure pump, a retentate tank, and a permeate tank [25]. The effective area of the RO membrane was 0.1 m^2^. The initial water tank was filled with a 1.5% wt.% HAc aqueous solution and the device was kept at a constant temperature of 30 °C during the experiment. Before the experiment, the RO membrane was cleaned with DI water at a flow rate of 1.5 L·min^−1^ for half an hour at an operating pressure of 1.5 MPa. Then, as the experiment proceeded, the HAc was concentrated into the retention tank. The RO permeate flux (*J_RO_*), concentration factor *(CF_RO_*), removal rate, and energy consumption (*E_RO_*) were evaluated as the operating pressure varied from 3.0 MPa to 5.0 MPa.

The RO permeate flux *(J_RO_*), concentration factor (*CF_RO_*), and energy consumption (*E_RO_*) were evaluated as the operating pressure varied from 3.0 MPa to 5.0 MPa.

The RO permeate flux *J_RO_* (L·m^−2^·h^−1^) was calculated by the following equation:

(1) $$J_{RO}=\frac{Q}{At}$$

where *Q* (L) represents the liquid volume of the permeate, *A* (m^2^) is the effective membrane area, and *t* (h) is the operation time.

The concentration factor *CF_RO_* was defined as in the following equation:

(2) $$CF_{RO}=\frac{C_{t}}{C_{0}}$$

where *C_t_* (wt.%) is the HAc concentration in the retentate at time *t* while *C*_0_ (wt.%) is the initial HAc concentration.

The energy consumption of RO *E_RO_* (kW·h·kg^−1^) was calculated by the following equation:

(3) $$E_{RO}=\frac{AP\times Q_{f}}{3.6\times 10^{6}\times m_{b}}$$

where Δ*P* (MPa) represents the difference between the pressure of raw wastewater and the feed pressure at the entrance of the membrane, *Q_f_* (L·h^−1^) represents the feed flow rate, and *m_b_* (g) represents the mass of HAc transferred in an hour.

### 2.3. ED Experiment

**Figure 2:**
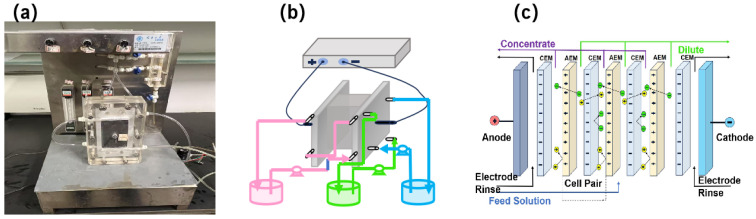
The experimental equipment of ED, assembled at Tianjin University. (**a**) The schematic diagram of (**b**) lab−scale ED configuration and ED cell (**c**).

The laboratory device was designed and assembled of ED as shown in Figure 2. The lab-scale electrodialysis configuration included a direct current power (GW instek, Suzhou, China), electrodialysis membrane stack, pumps, and solution tanks assembled as shown in Figure 1. Three streams were pumped from three tanks into different chambers with recirculation. There were 10 pairs of membranes in the ED stack and the effective area of each membrane was 84 cm^2^. Titanium plates were used as both anode and cathode. The electrode voltage was 8–16 V.

Before the experiment, 500 mL of aqueous HAc solution was introduced into the dilute and concentrated chamber, while 2000 mL of 0.2 mol/L H_2_SO_4_ solution [20] served as the electrode-washing liquid. The solution in the tank was pumped into the compartments within the membrane stack and then returned to their respective tanks.

The flow rates of the dilute stream, concentrate stream, and electrode solution were maintained at a constant temperature (30 °C). Subsequently, upon reaching a stable and bubble-free outflow from the membrane stack, the direct current power supply was switched on. The HAc concentration in the concentrated chamber increased and reached equilibrium and then the experiment was terminated.

Samples of 0.1 μL were taken at 10-min intervals from both the dilute and concentrated solutions, and the HAc concentration was determined using gas chromatography (GC) with a flame ionization detector (FID) and a capillary column (KB-FFAP, Beijing Beifen-Ruili Analytical Instrument (Group) Co., Ltd., Beijing, China, 30 m × 0.32 mm × 0.5 μm) [26]. Based on the distinctive characteristics and peak position of HAc, ethanol was selected as the internal standard [27]. The ED permeate flux (*J_ED_*), concentration factor (*CF_ED_*), current efficiency, water recovery ratio, and energy consumption (*E_ED_*) were evaluated.

In ED processes, the average flux *J_ED_* (g·m^−2^·h^−1^·CP^−1^) of HAc was calculated by the following equation.

The concentration factor *CF_ED_* was defined in the following equation:

(4) $$J_{ED}=\frac{m_{a}}{nAt}$$

where *m_a_* (g) represents the mass of HAc transported from the dilute chamber to the concentrated chamber, *A* (m^2^) is the effective membrane area, *n* represents the number of repeating units, and *t* (h) is the operation time.

The concentration factor *CF_ED_* was defined as in the following equation:

(5) $$CF_{ED}=\frac{C_{t}}{C_{0}}$$

where *C_t_* is the HAc concentration in the concentrated solution at time *t* and *C*_0_ is the initial HAc concentration.

The following equation calculated the current efficiency (%):

(6) $$\eta =\frac{z(C_{t}m_{t}-C_{0}m_{0})F}{NMI}$$

where *C*_0_ and *C_t_* are the concentrations of HAc at time 0 and *t*, *m*_0_ and *m_t_* indicate the masses of solution in the concentrated chamber at time 0 and *t*, *F* represents the Faraday constant (96,485 C/mol), *N* represents the number of repeating units, *M* represents the relative molecular mass of HAc, and *I* represents the current applied.

The following equation calculated the water recovery ratio:

(7) $$R=\frac{V_{dt}}{V_{d0}}$$

where *V_d_*_,*t*_ (mL) indicates the volume of solution in the dilute chamber at time *t* while *V_d_*_,0_ (mL) indicates the initial volume of solution in the dilute chamber.

The energy consumption of ED (kW·h·kg^−1^) was calculated by the following equation:

(8) $$E_{ED}=\frac{\int_{0}^{t}\;UIdt}{m_{a}}$$

where *U* (V) is the voltage of ED, *I* (A) is the current of ED, *t* is the time, and a denotes the mass of aqueous HAc solution transported from the dilute chamber to the concentrated chamber.

The enrichment percentage was defined as in the following equation:

(9) $$E_{e}=\frac{m_{L}}{m_{0}}\times 100\%$$

where *E_e_* is the enrichment percentage, *m_L_* denotes the mass of aqueous HAc solution transported, and *m*_0_ means initial HAc mass.

### 2.4. Analytical Methods

#### 2.4.1. Acid Concentration Analyses by Gas Chromatography

Samples of 0.1 μL were taken at 10-min intervals from both the dilute and concentrated solutions, and the HAc concentration was determined using gas chromatography (GC) with a flame ionization detector (FID) and a capillary column (KB-FFAP, Beijing Beifen-Ruili Analytical Instrument (Group) Co., Ltd., Beijing, China, 30 m × 0.32 mm × 0.5 μm) [27]. Based on the distinctive characteristics and peak position of HAc, ethanol was selected as the internal standard.

#### 2.4.2. Membrane Characterization

The changes in the membranes before use and after 90 days (about 1200 h) of use were investigated, and the ion exchange capacity (*IEC*) and migration number (t) of AEM and CEM were determined using the methods reported in the literature [4,27].

## 3. Results and Discussion

### 3.1. Comparison of RO and ED Processes for HAc Enrichment

#### 3.1.1. Effect of Operating Pressure on RO

This section investigates the impact of operating pressure on the permeate flux and concentration rate. The feed flow rate of the solution was 120 L/h, the HAc concentration was 1.5 wt.%, the operating pressures were set at 2.5, 3.0, 3.5, 4.0, 4.5, and 5.0 MPa, and the experimental temperature was 30 °C.

**Figure 3:**
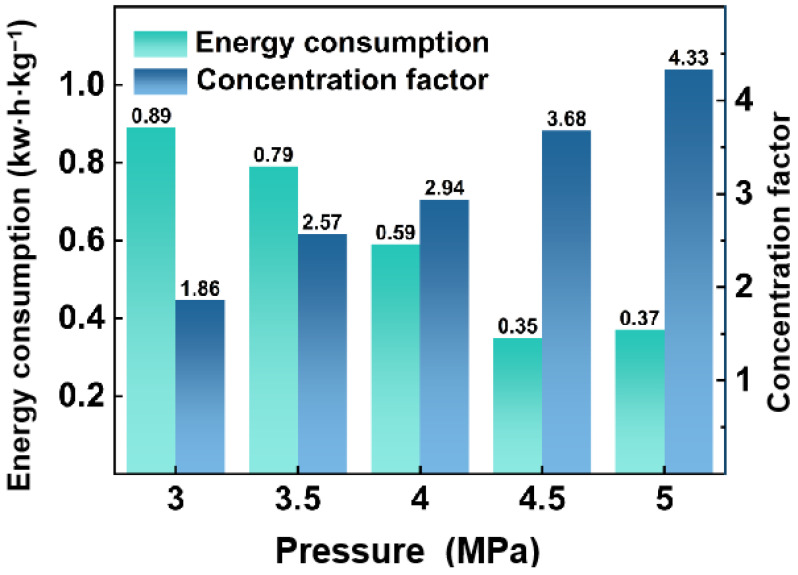
Effect of operating pressure on energy consumption and concentration factor of RO (feed HAc concentration of 1.5 wt.% with a flow rate of 120 L/h, 3 MPa to 5.0 MPa operating pressure, respectively, 0.7 reflux ratio, at 30 °C).

Figure 3 depicts the concentration factor and energy consumption of the RO process. An increase in operating pressure from 3 to 5 MPa boosts the permeate flux from 65.38 to 102.58 L·m^−2^·h^−1^. Hydrophilic polyamide membranes exhibit a stronger affinity for water than HAc. The enhanced retention capacity of acetic acid under a higher operating pressure significantly improves the separation efficiency between acetic acid and water. The applied pressure is raised from 3 to 5 MPa and the HAc concentration factor (CF) rises from 1.86 to 4.33. This improvement in CF significantly reduces energy consumption, with the power requirements dropping from 0.89 kW·h·kg^−1^ to 0.37 kW·h·kg^−1^ as the pressure is elevated within the same range.

#### 3.1.2. The Effect of Voltage on ED

The operating voltage is vital for the enrichment performance of HAc. Here, the constant voltage drop mode was adopted to study the effect of operating voltage. The operating voltages were set at 8 V, 10 V, 12 V, 14 V, and 16 V. The raw water flow rate of the HAc solution was 30 L/h. The HAc concentration was 1.5 wt.%. The experimental temperature was maintained at 30 °C.

**Figure 4:**
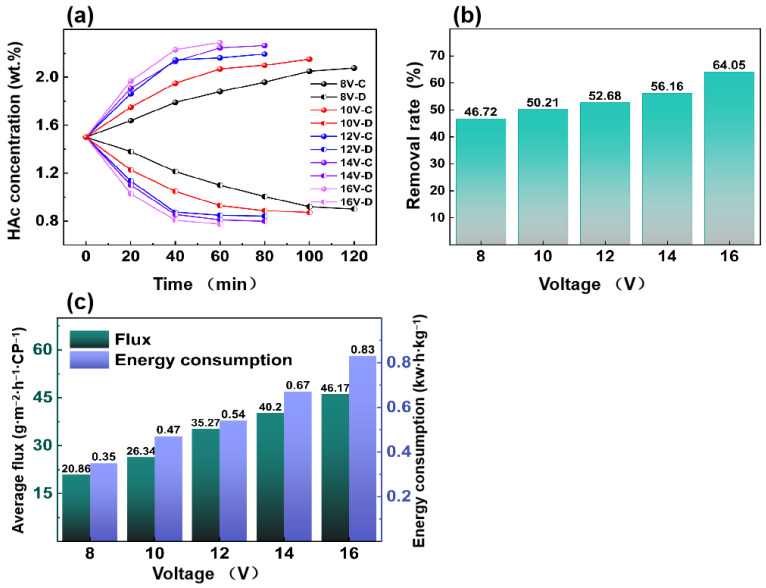
Effect of operating voltage on (**a**) HAc concentration, enrichment percentage, (**b**) and HAc average flux and energy consumption (**c**) (feed HAc concentration of 1.5 wt.% with a flow rate of 30 L/h; volume ratio of the concentrated chamber to the dilute chamber is 1:1).

Figure 4a depicts the change in HAc concentration in the concentrated and dilute solution in ED experiments when the voltage ranges from 8 to 16 V. When the voltage changes, the total operation time decreases from 120 min to merely 60 min. A higher applied voltage leads to a stronger driving force, resulting in a shorter experimental time. Notably, the final HAc concentrations in the concentrated chambers reach approximately 2 wt.% at 12 V, 14 V, and 16 V.

Figure 4b depicts the concentration percentage in ED experiments when the voltage ranges from 8 to 16 V. With the increase in voltage, the enrichment percentage increases from 46.72% to 64.05%.

Figure 4c depicts the relationship between voltage and the average flux of HAc/energy consumption. As the voltage rises from 8 to 16 V, the average flux of HAc increases from 20.86 g·m^−2^·h^−1^·CP^−1^ to 46.17 g·m^−2^·h^−1^·CP^−1^. Moreover, the energy consumption increases with a higher voltage, from 0.35 kW·h·kg^−1^ to 0.83 kW·h·kg^−1^.

In the experiment, we optimized the operating voltage to 1.2 V/CP, at which only sporadic bubbles were observed. At 1.6 V/CP, the operating current exceeded the limiting current density, and bubbles were seen at the electrode due to the production of H_2_ and O_2_. During the experiment, bubbles were also clearly seen at the electrode under the operating condition of 1.6 V/CP.

#### 3.1.3. Comparison of Energy and Cost for HAc Enrichment

This section compares the energy consumption of RO and ED under identical concentration factors.

**Figure 5:**
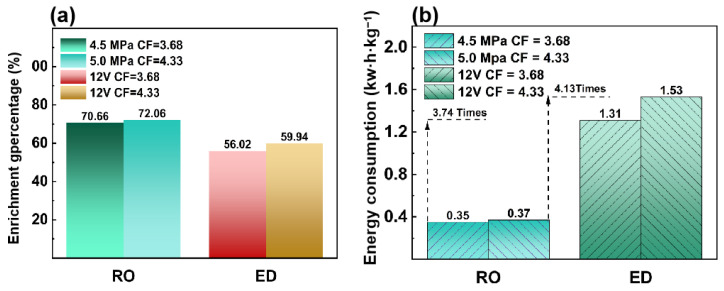
Enrichment percentage of RO and ED process. (**a**) Comparison of energy consumption between the RO process (feed HAc concentration of 1.5% with a flow rate of 120 L/h, 4.5 MPa, 5.0 Pa operating pressure respectively at 30 °C) and ED process (feed HAc concentration of 1.5% with a flow rate of 30 L/h, 12 V) (**b**).

Figure 5 illustrates the energy consumption between the RO and ED processes. In the RO process, when operating at 4.5 MPa, the enrichment percentage is 70.66% and the energy consumption remains constant at 0.35 kW·h·kg^−1^. When operating at 5.0 MPa, the enrichment percentage is 72.06%, and the energy consumption is 0.37 kW·h·kg^−1^ with a concentration factor of 4.33. In the ED process, when the concentration factor is 3.68, the enrichment percentage is 56.02% and the energy consumption is 1.31 kW·h·kg^−1^. When the concentration factor is 4.33, the enrichment percentage is 59.94% and the energy consumption is 1.53 kW·h·kg^−1^.

Table 4 shows the cost of RO and ED processes when HAc is concentrated from 1.5 wt.% to 6.5 wt.%. It can be seen that the price of RO is less than one-third of the price of ED. For low-concentration HAc solutions, RO is more energy-efficient than the ED process. Therefore, in the coupled RO–ED process, we first used the RO, followed by the ED, to enrich HAc.

### 3.2. Operation Mode in the ED Process

#### 3.2.1. Effect of Volume Ratio in the First ED Process

The volume ratio between the dilute chamber and the concentrated chamber plays an important role in the concentration of HAc product in the concentrated chamber. After the RO process, the HAc concentration was 6.5%, which was used as the feed directly for the ED process. In the ED process, the solution volume of the concentrated chamber was constant at 500 mL, and the solution volumes of the dilute chamber were 500 mL, 1000 mL, 1500 mL, 2000 mL, or 2500 mL.

**Figure 6:**
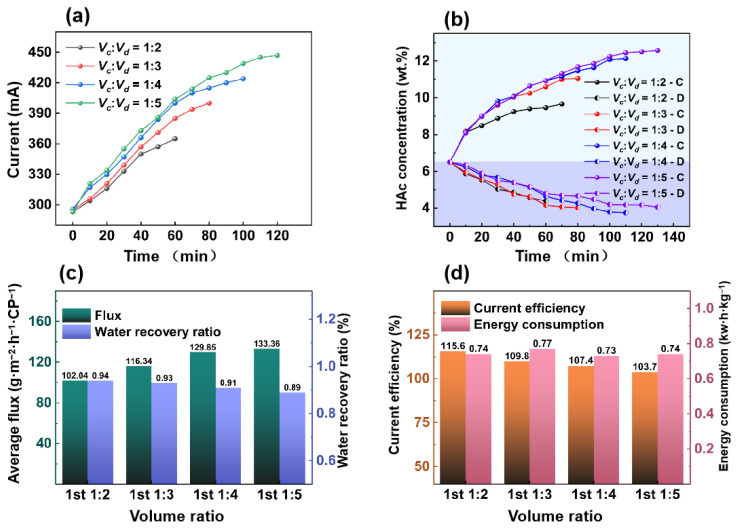
The first ED process under volume ratios. (**a**) Current vs. time. (**b**) HAc concentration vs. time. (**c**) Average flux of HAc/water recovery ratio vs. volume ratios. (**d**) Current efficiency/energy consumption vs. volume ratios. (feed HAc concentration of 6.5% with a flow rate of 30 L/h, 12 V).

Figure 6a shows that as the volume ratio of the concentrated chamber to the dilute chamber changes from 1:1 to 1:5, the current also increases. This phenomenon is because the conductivity of the concentrated chamber increases faster than that of the dilute chamber, resulting in a decrease in the electrodialysis resistance [30].

Figure 6b shows the change in HAc concentration in the concentrating and dilute chamber. When the volume ratio changes from 1:2 to 1:5, the HAc concentration in the concentrated chamber increases from 8.2 wt.% to 12.56 wt.%.

Figure 6c shows the relationship between HAc flux and volume ratio. At a volume ratio of 1:5, the HAc flux reaches 133.36 g·m^−2^·h^−1^·CP^−1^. When the volume ratio changes from 1:1 to 1:5, the water recovery rate decreases from 0.94 to 0.89.

Figure 6d shows the trend of current efficiency and energy consumption. When the volume ratio is 1:1, the current efficiency is at 115.6%. At a volume ratio 1:4, the minimum energy consumption is 0.73 kW·h·kg^−1^. A large solution volume of the dilute chamber increases the concentration efficiency but requires a higher current and longer run time. The current efficiency is also greater than 100%. That is because the HAc concentration in the feed solution is as high as 6.5 wt.%. Due to hydrogen bonding, the acetic acid molecules and acetate ions are combined and migrate to the concentrated chamber.

#### 3.2.2. Effect of Volume Ratio in the Second ED Process

The first-stage ED process achieved a concentration factor of 1.92 with a volume ratio of 1:4, requiring a secondary ED for a higher HAc concentration. The secondary ED operating voltage was 12 V and the volume ratios were 1:4 or 1:5. The feed HAc concentration was 12.5 wt.%.

**Figure 7:**
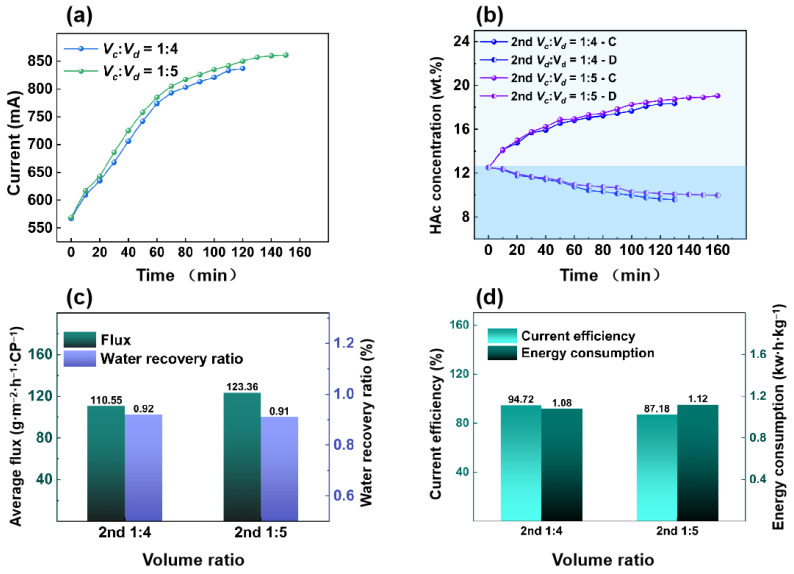
The second ED process under volume ratios. (**a**) Current vs. time. (**b**) HAc concentration vs. time. (**c**) Average flux of HAc/water recovery ratio vs. volume ratios. (**d**) Current efficiency/energy consumption vs. volume ratios. (feed HAc concentration of 12.5 wt.% with a flow rate of 30 L/h, 12 V).

Figure 7a shows the relationship between the volume ratio and the current. When the volume ratio is 1:4 and 1:5, the current is 837 mA and 860 mA, respectively. As shown in Figure 7b, when the volume ratio is 1:4 and 1:5 in the second ED process, the final HAc concentration in the concentrated chamber is 18.35% and 19.02%, respectively. When the volume ratio is 1:5, the concentration ratio of the second ED is 1.52, which is lower than that of the first ED (1.92). As shown in Figure 6c, when the volume ratio is 1:4 and 1:5, the flux of HAc is 110.55 g·m^−2^·h^−1^·CP^−1^ and 123.36 g·m^−2^·h^−1^·CP^−1^, respectively. The flux of the second ED process exceeds that of the first ED process because the increase in conductivity reduces the membrane stack’s resistance. At the same time, the water recovery rate dropped from 0.92 to 0.91. As shown in Figure 7d, when the volume ratio is 1:4, the energy consumption is 1.08 kW·h·kg^−1^ and the current efficiency is 94.72%. In contrast, when the volume ratio is increased to 1:5, the energy consumption is slightly higher, 1.12 kW·h·kg^−1^, but the current efficiency is significantly reduced to 87.18%.

#### 3.2.3. The Stability of Membrane

The changes in the membranes before use and after 90 days (about 1200 h) of use were listed in Table 5. The migration number of the CEM decreased from 0.98 to 0.96, and the migration number of the AEM decreased from 0.93 to 0.90, indicating that the selectivity of the ion exchange membranes decreased [31]. After use, the ion exchange capacity (IEC) of the CEM and AEM decreased by 12.72% and 16.51%, respectively. The surface resistance of the CEM increased from 3.3 Ω·cm^−2^ to 3.6 Ω·cm^−2^, and the surface resistance of the AEM increased from 2.0 Ω·cm−^2^ to 2.4 Ω·cm^−2^. The increase in resistance led to an increase in the transmembrane resistance of ion migration, reducing the ED efficiency [32].

## 4. Conclusions

This study investigated the lab-scale integration of RO and ED for acid wastewater treatment, demonstrating the integration process’s technical feasibility and energy advantages. We studied the effects of operating pressure in the RO process and the effects of parameters, including voltage and volume ratio, in the two-stage ED processes. At 30 °C, for a low-concentration HAc solution of 1.5 wt.%, we first used RO to concentrate the HAc to 6.5 wt.%, with an energy consumption of 0.37 kW·h·kg^−1^. Subsequently, the HAc was concentrated to 12.5 wt.% in the first ED process, with an energy consumption of 0.73 kW·h·kg^−1^. In the second ED process, the HAc was concentrated to 19.0 wt.%, with an energy consumption of 1.12 kW·h·kg^−1^. The enriched HAc can be used in the subsequent distillation process. This paper provides a promising and sustainable strategy for enriching low-concentration acids.

## Figures and Tables

**Table 1:** The main characteristics of the RO membrane used in these experiments.

	SW30-4040
Separation layer	Polyamide membrane
pH	2~11
Temperature (°C)	10~40
Maximum operating pressure (MPa)	6.5
Maximum feed flow rate (L·h^−1^)	3600

**Table 2:** The main characteristics of CEM and AEM used in these experiments.

	CEM (CT-4)	AEM (ATD)
Thickness (μm)	70~80	70~8 0
Water content (wt.%)	20~25	20~25
Transport number	≥0.98	≥0.97
IEC (mmol·g^−1)^	0.9~1.1	0.9~1.1
Area electric resistance (Ω·cm^2^)	3.5~4.0	4.0~5.5
pH	0~14	0~14
Temperature (°C)	15~40	15~40

**Table 3:** The main characteristics of EM used in these experiments.

	DMR100
Thickness (μm)	75
Tensile strength (transverse/longitudinal) (MPa)	≥30/30
Elongation at break (transverse/longitudinal) (MPa)	≥100/100
Electrical conductivity (mS/cm)	≥30
Chemical durability/h (90 °C, 30% RH)	800
Hydrogen permeability (mL/min cm^2^)	≤0.01

**Table 4:** The cost of RO and ED processes when HAc is concentrated from 1.5 wt.% to 6.5 wt.% (RO operating energy consumption for peripheral equipment is calculated in the use of energy consumption, the calculation formula for the cost is detailed in S1 [24,28,29].)

	Cost (ED)	Cost (RO)
	**Energy cost**	**Energy cost**
Energy consumption (kW h/kg)	1.53	0.37
Electricity charge (CNY/(kW h))	0.70	0.70
Energy consumption for peripheral equipment(pump) (CNY/kg)	0.14	/
Total energy cost (CNY/kg)	0.067	0.015
	**Investment cost**	**Investment cost**
Membrane life (year)	3	2
Membrane price (CNY/m^2^)	1515.14	320
Membrane cost (CNY)	254.54	45.71
Membrane stack cost (CNY)	381.81	68.56
Peripheral equipment cost (CNY)	572.72	137.13
Total fixed cost (CNY/year)	318.18	102.85
Total fixed cost (CNY/kg)	0.18	0.058
Total process cost (CNY/kg)	0.25	0.069

**Table 5:** The results of ED performance of AEM and CEM.

	CEM		AEM	
ED properties	Before use	After use	Before use	After use
Transport number	0.98	0.96	0.93	0.90
Ion exchange capacity (mmol·g^−1^)	1.10	0.96	1.09	0.91
Area electric resistance (Ω·cm^2^)	3.3	3.6	2.0	2.4

## Data Availability

The original contributions presented in the study are included in the article; further inquiries can be directed to the corresponding authors.

## Supplementary Materials

The following supporting information can be downloaded at: https://www.mdpi.com/article/10.3390/membranes15050129/s1, S1: The energy consumption cost for peripheral equipment (CNY/kg) (S1); The total energy cos (CNY/kg) (S2); The membrane cost (CNY) (S3); The membrane stack cost (CNY) (S4); The peripheral equipment cost (CNY) (S5); The total fixed cost (CNY/year) (S6); The Total fixed cost* (CNY/year) (S7); The Total process cost (CNY/kg) (S8).
