# Supplementary material for: The Enrichment of Acetic Acid Using an Integrated Reverse Osmosis–Electrodialysis Process
Source: Membranes (Basel). 2025 Apr 27;15(5):129. doi: 10.3390/membranes15050129 (PMC12113363; doi:10.3390/membranes15050129)
Supplement: Supplementary file 1 [file membranes-15-00129-s001.zip › membranes-3566941-supplementary.pdf]

membranes

MDPI

Supplementary Materials

The enrichment of Acetic Acid Using an Integrated Reverse Osmosis-Electrodialysis Process

Shichang Xu [1,2], Long Zhang [1,2], Zhen Zhang [1,2], Lixin Xie [1,2,*] and Wen Zhang [1,2,*]

1 State Key Laboratory of Chemical Engineering and Low-Carbon Technology, School of Chemical Engineering and Technology, Tianjin University, Tianjin 300350, China
2 Tianjin Key Laboratory of Membrane Science & Desalination Technology, School of Chemical Engineering and Technology, Tianjin University, Tianjin 300350, China
xu_sc1@tju.edu.cn (S.X.); zhanglong123@tju.edu.cn (L.Z.); zhangzhen123@tju.edu.cn (Z.Z.);
* Correspondence: xie_lixin@tju.edu.cn (L.X.); zhang_wen@tju.edu.cn (W.Z.)

S1

The energy consumption cost for peripheral equipment Cp (CNY/kg) was calculated by the following equation,

$$C_p = \zeta \cdot E_{ED} \cdot E_C \quad \text{(S1)}$$

, where ζ is the is the correction factor, ζ_{ED} = 0.2,. $E_{ED/RO}$ is the energy consumption of ED, E_C is the average price of industrial electricity, 0.7 CNY/KWh[24].

The total energy cost TEC(CNY/kg) was calculated by the following equation,

$$TEC = m_p \cdot (E_{ED/RO} + C_p) \cdot E_C \quad \text{(S2)}$$

, where m_p is the acid migration mass per kilogram of product produced, $E_{ED/RO}$ is the energy consumption of RO/ED. m_{pED} = 0.052 kg, m_{pRO} = 0.041 kg, E_C is the average price of industrial electricity, 0.7 CNY/KWh

The membrane cost M_c (CNY) was calculated by the following equation,

$$M_c = A_E \cdot C_M \quad \text{(S3)}$$

, where A_E is the actual membrane area used, C_M is the price of membrane. A_{ERO} =0.135 m^2, A_{EED} = 0.168 m^2, C_{MRO}= 1515 CNY/m^2, C_{MED} = 320 CNY/m^2

The membrane stack cost M_{SC} (CNY) was calculated by the following equation,

$$M_{sc} = \kappa \cdot M_c \quad \text{(S4)}$$

, where κ is thehe correction factor of membrane stack cost κED = 1.5, κRO = 2.0, Mc is the membrane cost[24]. M_{cRO} = 68.56 CNY, M_{cED}= 381.81 CNY[24,28].

The peripheral equipment cost (CNY) was calculated by the following equation,

$$C_{pe} = \eta \cdot M_{sc} \quad \text{(S5)}$$

, where η is thehe correction factor of peripheral equipment cost η = 1.5, M_{sc} is the membrane stack cost[24]. ηED = 1.5, ηRO = 2.0,. M_{cRO} = 137.13, CNY, M_{cED}= 572.7 CNY[24,29].

The total fixed cost TFC (CNY/year) was calculated by the following equation,

$$TFC = \frac{M_c + M_{sc}}{M_L} \quad \text{(S6)}$$

, where M_c is the membrane cost, M_{sc} is the peripheral equipment cost, M_L is the membrane life (which is presented in table 5).

The Total fixed cost* TFC* (CNY/year) was calculated by the following equation,

$$TFC^* = \frac{TFC}{P_{Annual}} \quad \text{(S7)}$$

, where TFC is the total fixed cost, P_{Annual} is the annual production measured by laboratory equipment. P_{ED} = 2064.3 kg, P_{RO} = 2487.3 kg.

The Total process cost *TPC* (CNY/kg) was calculated by the following equation,

$$TPC = TEC + TFC^* \quad \text{(S8)}$$

, where TFC is total fixed cost and TFC^*. P_{Annual} is the annual production measured by laboratory equipment. P_{ED} = 2064.3kg, P_{RO} = 2487.3 kg. TFC_{RO} = 0.015 CNY/kg, TFC^*_{RO}= 0.058 CNY/kg. TFC_{ED} = 0.067 CNY/kg, TFC^*_{ED} =0.18 CNY/kg.

References

24. Qiu, Y.; Ruan, H.; Tang, C.; Yao, L.; Shen, J.; Sotto, A. Study on Recovering High-Concentration Lithium Salt from Lithium-Containing Wastewater Using a Hybrid Reverse Osmosis (RO)–Electrodialysis (ED) Process. *ACS Sustain. Chem. Eng.* **2019**, *7*, 13481–13490.

28. Patel, S.K.; Lee, B.; Westerhoff, P.; Elimelech, M. The potential of electrodialysis as a cost-effective alternative to reverse osmosis for brackish water desalination. *Water Research* **2024**, *250*, 121009.

29. Filteau, G.; Moss, P. Ultra-low pressure RO membranes: an analysis of performance and cost. *Desalination* **1997**, *113*, 147-152.
